# Tick range expansion to higher elevations: does *Borrelia burgdorferi* sensu lato facilitate the colonisation of marginal habitats?

**DOI:** 10.1186/s12862-022-02058-x

**Published:** 2022-08-26

**Authors:** Mélissa Lemoine, Luca Cornetti, Kevin Reeh, Barbara Tschirren

**Affiliations:** 1 Department of Evolutionary Biology and Environmental Studies, University of Zurich, Winterthurerstrasse 190, 8057 Zurich, Switzerland; 2 Centre for Ecology and Conservation, University of Exeter, Penryn, TR10 9FE UK

**Keywords:** *Ixodes ricinus*, Parasite range expansion, Host phenotypic alterations, Host and vector manipulation by parasites, Lyme disease, *Borrelia burgdorferi* sensu lato, Global climate change warming, Elevational gradient

## Abstract

**Background:**

Parasites can alter host and vector phenotype and thereby affect ecological processes in natural populations. Laboratory studies have suggested that *Borrelia burgdorferi* sensu lato, the causative agent of human Lyme borreliosis, may induce physiological and behavioural alterations in its main tick vector in Europe, *Ixodes ricinus*, which increase the tick’s mobility and survival under challenging conditions. These phenotypic alterations may allow *I. ricinus* to colonise marginal habitats (‘facilitation hypothesis’), thereby fuelling the ongoing range expansion of *I. ricinus* towards higher elevations and latitudes induced by climate change. To explore the potential for such an effect under natural conditions, we studied the prevalence of *B. burgdorferi* s.l. in questing *I. ricinus* and its variation with elevation in the Swiss Alps.

**Results:**

We screened for *B. burgdorferi* s.l. infection in questing nymphs of *I. ricinus* (N = 411) from 15 sites between 528 and 1774 m.a.s.l to test if *B. burgdorferi* s.l. prevalence is higher at high elevations (i.e. in marginal habitats). Opposite of what is predicted under the facilitation hypothesis, we found that *B. burgdorferi* s.l. prevalence in *I. ricinus* nymphs decreased with increasing elevation and that *Borrelia* prevalence was 12.6% lower in *I. ricinus* nymphs collected at the range margin compared to nymphs in the core range. But there was no association between *Borrelia* prevalence and elevation within the core range of *I. ricinus*. Therefore the observed pattern was more consistent with a sudden decrease in *Borrelia* prevalence above a certain elevation, rather than a gradual decline with increasing elevation across the entire tick range.

**Conclusions:**

In conclusion, we found no evidence that *B. burgdorferi* s.l.-induced alterations of *I. ricinus* phenotype observed in laboratory studies facilitate the colonisation of marginal habitats in the wild. Rather, ticks in marginal habitats are substantially less likely to harbour the pathogen. These findings have implications for a better understanding of eco-evolutionary processes in natural host-parasite systems, as well as the assessment of Lyme borreliosis risk in regions where *I. ricinus* is newly emerging.

## Background

Parasites can alter the physiology, morphology and behaviour of their hosts [1–3]. Vectors can similarly be the target of parasite manipulation [4, 5]. Such manipulations can facilitate parasite transmission, and are thus adaptive from the parasite’s perspective [6]. However, not all phenotypic alterations are the result of such adaptive manipulations [7, 8]. Phenotypic changes in infected hosts and vectors can also be a side effect of infection that is of no adaptive value for the parasite.

Surprisingly little is known about the ecological consequences of parasite-induced phenotypic alterations in hosts or vectors. Recent studies have suggested that parasite-induced changes may have profound effects on host population dynamics, trophic niche specialisation as well as interactions between competitors and predators at all trophic levels [9–11]. Consequently, parasites can have a strong ecological impact that becomes especially apparent when they are missing or introduced, e.g. during biological invasions [11, 12]. Invasive host populations often harbour fewer parasites than populations in their native range, which may enhance their population growth and competitive ability (i.e. ‘enemy release hypothesis’ [13]). Parasites may be ‘lost’ because of low host densities and founder effects during the three phases of an invasion: introduction, establishment and range expansion [14]. However, empirical studies investigating the role of parasites during range expansion from core populations, i.e. without bottlenecks occurring during the introduction and establishment phases, are still rare [15], and both increasing and decreasing parasite loads at the host range margins have been reported (e.g. [16–18]).

The distribution of *Ixodes* spp. ticks is strongly influenced by abiotic factors, such as temperature and humidity, and there is accumulating evidence that in Europe and North America *Ixodes* spp. ticks have been expanding northwards as well as towards higher elevations during the last decades due to climate warming (reviewed in [19, 20]). In Europe, *I. ricinus* is the main vector of the bacterium *Borrelia burgdorferi *sensu lato, the causative agent of human Lyme borreliosis. *Borrelia burgdorferi* s.l. forms a complex comprising of at least 18 genospecies [21]. A diverse host community, which includes rodents, insectivores, birds and reptiles, acts as reservoir hosts for *B. burgdorferi* s.l.. In Europe, *B. afzelii* and *B. garinii* are the most common genospecies [22]. *Borrelia afzelii* is a rodent specialist, infecting mice (*Apodemus sylvaticus* and *A. flavicollis*) and voles (*Myodes glareolus* and *Microtus agrestis*), but also shrews (*Sorex araneus* and *S. minutus*; [23]), whereas *B. garinii* is a bird specialist, infecting a range of bird species such as *Turdus* spp., *Sturnus vulgaris*, *Sylvia* spp. and *Parus* *major* [21, 24].

*Ixodes ricinus* is a three host tick. Each life stage (i.e. larva, nymph and adult) seeks a host, feeds, and drops off to develop in the natural environment. Although *I. ricinus* is a host generalist, the host distribution differs across tick stages [25, 26]. The host selection is, among other things, driven by habitat type, host behaviour, microclimate conditions, and questing height of the different stages [26]. Adults quest higher in the vegetation than nymphs, and nymphs higher than larvae. Larvae and nymphs feed on vertebrate hosts of all size classes but particularly on rodents and birds, while adult females usually feed on larger mammals [26]. Unlike hematophagous insects, ticks typically feed for several days on their host, and the transmission of tick-borne pathogens such as *B. burgdorferi* s.l. to the host may start several hours after tick attachment [26].

Various studies have reported that bacteria and viruses may induce phenotypic alterations in *Ixodes* spp. [26, 27]. Specifically, laboratory studies have suggested that *B. burgdorferi* s.l.-infected *I. ricinus* are bigger, walk faster and longer, quest longer for a host, have higher energy reserves (for the same body mass) and survive better under challenging thermohygrometric conditions than uninfected *I. ricinus.* Yet to date, studies investigating such vector manipulation by pathogens remain scarce and results are heterogeneous ([26, 27], and references therein). These alterations of tick behaviour and physiology may benefit pathogen transmission by extending vector lifespan, or by increasing host-finding efficacy [26]. Importantly, both may facilitate the colonisation of marginal habitats by ticks, and lead to particularly high prevalence of *B. burgdorferi* s.l. in *I. ricinus* populations at range margins [28]. Particularly, higher fat contents in *Borrelia*-infected nymphs may provide an advantage under colder or more variable temperatures, such as at higher elevations, although laboratory data were not conclusive [29]. Furthermore, increased host-finding efficacy may facilitate the establishment of tick populations in less diverse or more fragmented host communities. Finally by modulating questing behaviour, such as adjusting questing height or moving to shade, *Borrelia* infection may shape the interaction between ticks and host species [26, 27]. Specifically, questing higher up in the vegetation may allow ticks to attach to larger animals. Since larger animals have larger home ranges [30], questing at higher heights may allow ticks to disperse further. All these possible mechanisms may result in higher *B. burgdorferi* s.l. prevalences in *I. ricinus* populations at range margins. Assessing the role of *B. burgdorferi* s.l. in influencing vector range expansion to marginal habitats is crucial for our understanding of eco-evolutionary processes in natural host-parasite systems, but also for quantifying public health threats in regions where ticks are newly emerging. Yet, to our knowledge no study has tested this hypothesis in *I. ricinus* populations under natural conditions to date.

Although exclusion experiments (i.e. where a taxon is excluded) are the gold standard to assess the ecological role of parasites, such manipulations remain an enormous practical and logistic challenge in *I. ricinus* and its pathogens. Moreover, experiments in the wild testing whether *B. burgdorferi* s.l. enhances survival and reproduction of *I. ricinus* under harsh conditions (i.e. physiological alterations) would only provide a partial picture because *B. burgdorferi* s.l. might affect dispersal abilities of *I. ricinus* through its ability to find a host (i.e. behavioural alterations). Because of these practical challenges, we used a correlational approach in naturally infected tick populations in the Swiss Alps to evaluate the potential of *B. burgdorferi* s.l. to facilitate the colonisation of marginal habitats by *I. ricinus*. Previous studies monitored *B. burgdorferi* s.l. and *I. ricinus* within their core range [31–36], at the range margins without a direct comparison with core populations [37] or when *B. burgdorferi* s.l. prevalence was low [38–40] (but see [41]). In our study we monitored tick populations at different elevations, including core range and range margin populations, allowing for a direct comparison of *Borrelia* prevalence across the tick distribution range. We predict that if *B. burgdorferi* s.l. alters the phenotype of *I. ricinus*, thereby making them better colonizers of unfavourable habitats, *B. burgdorferi* s.l. prevalence will be disproportionally high at the tick range margin (i.e. at high elevations).

## Results

### Questing *I. ricinus* abundance along elevation

**Fig. 1:**
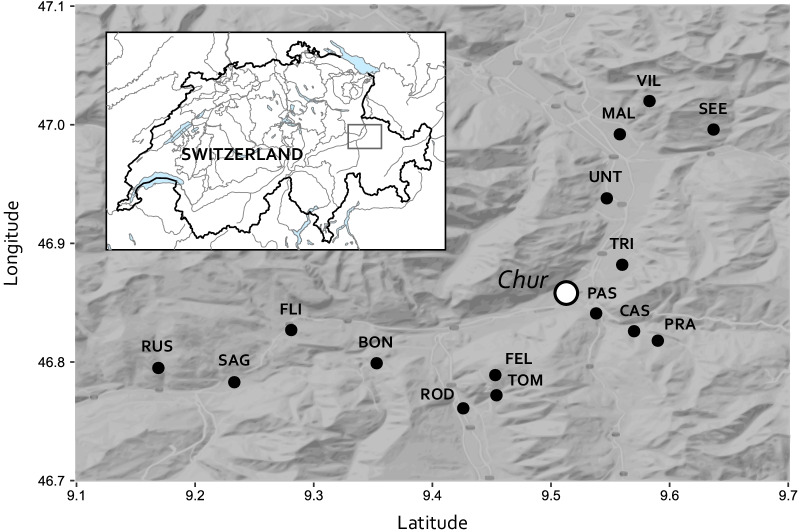
Location of the study sites in the Swiss Alps. Sites are indicated by black dots. Acronyms refer to the sites listed in Table 1

We collected a total of 1138 questing nymphs and 270 questing adults across three 1 h-dragging sessions at 15 sites each (Table 1; Fig. 1; 0–109 nymphs and 0–44 adults per dragging event, Table 2). Overall, we collected at least four times more nymphs than adults. Patterns of tick abundance along elevation did not vary between tick stages (Interaction stage × elevation^2^: *χ*^2^_1_ = 0.88, *P* = 0.349; Interaction stage × elevation: *χ*^2^_1_ = 3.17, *P* = 0.075). Therefore, nymphs and adults were pooled for subsequent analyses.

**Table 1:** *Borrelia burgdorferi* s.l. prevalence in questing nymphs at 15 sites in the Swiss Alps

Site	Label	GPS Coordinates		Elevation	Range	N adult	N nymph	N all	Ticks /h	Analysed	*Borrelia*	*Borrelia*	% nymphs
		North	East	(m.a.s.l.)	exp	*I. ricinus*	*I. ricinus*	*I. ricinus*		nymphs	*afzelii*	others	infected
Untevaz	UNT	46.938	9.547	528	C	11	63	74	24.7	34	10	2	35
Malans	MAL	46.992	9.558	560	C	10	48	58	19.3	35	4	2	17
Rodels	ROD	46.761	9.426	630	C	13	63	76	25.3	34	10	4	41
Sagogn	SAG	46.783	9.233	693	C	57	19	76	25.3	35	8	2	29
Passug	PAS	46.841	9.538	732	C	56	241	297	99	34	9	0	26
Trimmis	TRI	46.882	9.56	762	C	35	271	306	102	35	6	4	29
Bonaduz	BON	46.799	9.353	944	C	33	68	101	33.7	32	6	0	19
Castiel	CAS	46.826	9.57	1094	C	17	116	133	44.3	34	4	2	18
Seewis	SEE	46.996	9.637	1106	C	18	88	106	35.3	35	8	0	23
Flims	FLI	46.827	9.281	1138	C	4	20	24	8	35	3	0	9
Tomils	TOM	46.772	9.454	1144	C	13	102	115	38.3	34	15	1	47
Ruschein	RUS	46.795	9.169	1454	M	2	26	28	9.3	22	1	0	5
Praden	PRA	46.818	9.59	1582	M	1	11	12	4	9	0	0	0
Feldis	FEL	46.789	9.453	1673	M	0	2	2	0.7	3	0	0	0
Vilan	VIL	47.02	9.583	1774	O	0	0	0	0	0	–	–	–

Sampling site and acronym, GPS coordinates, elevation, number of *I. ricinus* adults and nymphs collected over the three sampling sessions as well as the average tick abundance per hour (Tick/h) and tick range position of the site [range core (C), range margin (M) and outside of the range (O)] are given. Furthermore, the number of nymphs screened for *Borrelia* infection (analysed nymphs), the number of nymphs infected with *B. afzelii* and other *B. burgdorferi* s.l. genospecies and the infection prevalence (%) considering all *B. burgdorferi* s.l. genospecies are reported

**Table 2:** Questing tick abundance and *Borrelia burgdorferi* s.l. prevalence by sampling session and site

Sites	Questing tick abundance						*Borrelia* screening											
	Adults/h			Nymphs/h			Analysed nymphs				*Borrelia* s.l. occurrence				*Borrelia afzelii* occurrence			
	1	2	3	1	2	3	1	2	3	Total	1	2	3	Total	1	2	3	Total
UNT	7	4	0	46	12	5	16	13	5	34	7	3	2	12	5	3	2	10
MAL	7	2	1	11	32	5	11	13	11	35	2	2	2	6	1	1	2	4
ROD	5	8	0	42	11	10	11	11	12	34	4	4	6	14	3	2	5	10
SAG	44	5	8	7	7	5	12	11	12	35	6	2	2	10	4	2	2	8
PAS	29	22	5	98	108	35	12	11	11	34	3	2	4	9	3	2	4	9
TRI	12	12	11	109	63	99	14	12	9	35	6	2	2	10	4	1	1	6
BON	14	11	8	38	10	20	12	10	10	32	4	1	1	6	4	1	1	6
CAS	6	8	3	50	55	11	12	11	11	34	3	2	1	6	2	1	1	4
SEE	2	12	4	7	77	4	11	12	12	35	2	4	2	8	2	4	2	8
FLI	4	0	0	13	4	3	17	8	10	35	1	1	1	3	1	1	1	3
TOM	2	8	3	38	45	19	12	11	11	34	3	6	7	16	2	6	7	15
RUS	1	0	1	13	9	4	10	8	4	22	1	0	0	1	1	0	0	1
PRA	1	0	0	1	9	1	0	8	1	9	-	0	0	0	-	0	0	0
FEL	0	0	0	1	1	0	1	2	0	3	0	0	-	0	0	0	-	0
VIL	0	0	0	0	0	0	0	0	0	-	-	-	-	-	-	-	-	-

Sampling site acronym, number of *I. ricinus* adults and nymphs collected in the vegetation, the number of nymphs screened for *Borrelia* infection (analysed nymphs), the number of nymphs infected with *B. afzelii* and other *B. burgdorferi* s.l. genospecies during the three sampling sessions (in June, July and August)

**Fig. 2:**
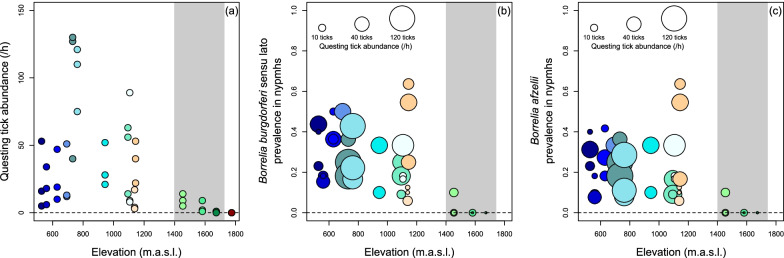
Tick abundance and *Borrelia* prevalence along elevation in the Swiss Alps. Abundance of questing *Ixodes ricinus* ticks in the vegetation (**a**) and prevalence of *Borrelia burgdorferi* sensu lato (**b**) and *Borrelia afzelii* (**c**) in *I. ricinus* nymphs at different elevations (m.a.s.l.) in the Swiss Alps. The colours of the dots represent the 3 dragging events per site while the size of the dots in (**b**) and (**c**) is proportional to the abundance of questing I. ricinus ticks (**a**). The grey area represents the range margin of *I. ricinus* ticks

Questing *I. ricinus* abundance decreased non-linearly with increasing elevation with an abundance peak at intermediate elevations (Elevation^2^: *χ*^2^_1_ = 17.62, *P* < 0.001, *β*_*2*_ = − 1.057 ± 0.206; Elevation: *χ*^2^_1_ = 17.06, *P* < 0.001, *β*_*1*_ = − 0.903 ± 0.170, Fig. 2a). No evidence for seasonal differences in tick abundance across elevations was found (Nymphs: Interaction session × elevation^2^: *χ*^2^_1_ = 0.88, *P* = 0.645; Interaction session × elevation: *χ*^2^_1_ = 2.95, *P* = 0.229; nymphs and adults pooled: Interaction session × elevation^2^: *χ*^2^_1_ = 1.55, *P* = 0.461; Interaction session × elevation: *χ*^2^_1_ = 3.28, *P* = 0.194).

Questing *I. ricinus* abundance was high up to an elevation of 1100 m.a.s.l., and then rapidly decreased (Table 1, Fig. 2a). No questing *I. ricinus* were found at the highest elevation site (VIL) at 1680 m.a.s.l.. We therefore defined sites below 1150 m.a.s.l. as the core range and sites between 1450 and 1680 m.a.s.l. to represent the elevational range margin of *I. ricinus* ticks in our study area (note that no sites between 1150–1450 m.a.s.l. were included in our study). The highest elevation site (VIL) was excluded from subsequent analyses because it is not yet colonised by ticks (i.e. outside the tick range). Questing tick abundance was higher in the core range than in the elevational range margin (Range: *χ*^2^_1_ = 13.44, *P* < 0.001, excluding VIL, Fig. 2a).

### *Borrelia* infection in *I. ricinus* nymphs across the tick range

Overall, 24.6% of the analysed *I. ricinus* nymphs (101 out of 411) were infected with *B. burgdorferi* s.l.. 83.2% of these infections were caused by *B. afzelii* (Table 1). Prevalence among sites varied between 0 and 47.1% for *B. burgdorferi* s.l., and 0–44.1% for *B. afzelii* (Table 1).

*Borrelia burgdorferi* s.l. prevalence decreased with increasing elevation (Elevation^2^: *χ*^2^_1_ = 3.10, *P* = 0.078; Elevation: *χ*^2^_1_ = 6.38, *P* = 0.011, *β* = − 0.664, Fig. 2b), whereas for *B. afzelii* the decrease with increasing elevation was marginally non-significant (Elevation^2^: *χ*^2^_1_ = 3.19, *P* = 0.074; Elevation: *χ*^2^_1_ = 3.39, *P* = 0.066; Fig. 2c). *Borrelia* prevalence was 12.6 and 9.8 times higher in *I. ricinus* nymphs collected within the core range than at the range margin for *B. burgdorferi* s.l. (*χ*^2^_1_ = 9.55, *P* = 0.002, Fig. 2b) and *B. afzelii* (*χ*^2^_1_ = 7.56, *P* = 0.006, Fig. 2c), respectively.

If abiotic factors underlie the decrease of *Borrelia* prevalence with increasing elevation across the entire tick range, we would predict to see a gradual decline of *B. burgdorferi* s.l. prevalence also within the core range of ticks. However, we found no evidence that *Borrelia* prevalence declined with elevation within the core range of ticks (*B. burgdorferi* s.l.: Elevation^2^: *χ*^2^_1_ = 0.07, *P* = 0.786; Elevation: *χ*^2^_1_ = 0.81, *P* = 0.369; *B. afzelii*: Elevation^2^: *χ*^2^_1_ = 0.13, *P* = 0.718; Elevation: *χ*^2^_1_ = 0.05, *P* = 0.823). Moreover, within the core range of ticks, no association between *Borrelia* prevalence and questing *I. ricinus* abundance was found for *B. burgdorferi* s.l. (Questing tick abundance^2^: *χ*^2^_1_ = 2.19, *P* = 0.1439; Questing tick abundance: *χ*^2^_1_ = 1.85, *P* = 0.174, Fig. 2b) or *B. afzelii* (Questing tick abundance^2^: *χ*^2^_1_ = 0.57, *P* = 0.449; Questing tick abundance: *χ*^2^_1_ = 0.47, *P* = 0.493, Fig. 2c).

## Discussion

Laboratory experiments have suggested that *B. burgdorferi* s.l. may increase tick tolerance to desiccation and boost host-finding efficacy ([26, 27], and references therein). Such vector manipulations by the pathogen may facilitate the range expansion of ticks to marginal habitats, such as higher elevations or higher latitudes [28]. Here we used a correlational approach to investigate whether patterns of *B. burgdorferi* s.l. prevalence in questing *I. ricinus* nymphs, and its variation with elevation, provide support for this hypothesis.

Overall, questing *I. ricinus* abundance was high and *B. burgdorferi* s.l. infection common in the study area: *I. ricinus* were found at 14 out of 15 sites, and *B. burgdorferi* s.l. infection in questing *I. ricinus* nymphs was detected at 12 sites. Above 1400 m.a.s.l., however, *I. ricinus* abundance strongly decreased and no *I. ricinus* were found above 1700 m.a.s.l.. *B. burgdorferi* s.l. prevalence in questing *I. ricinus* nymphs across sites was similar to previous reports from central Europe (e.g. [33], and references therein, [42]), but variation across sites was high (0–47% compared to 1–20% across Europe according to [22]), and it exceeded 40% at two sites of contrasting elevations (630 and 1123 m.a.s.l.).

As expected, the most common *B. burgdorferi* s.l. genospecies was *B. afzelii*, accounting for 83.2% of *I. ricinus* infections in our study. This is higher than what has been reported previously (7–68% of *I. ricinus* infections caused by *B. afzelii*; [22]) across Europe but similar to what has recently been reported from the Italian Alps ([41] where 73.1% of the nymphs were infected by *B. afzelii*). Differences across studies and locations are likely due to differences in the composition of the local host community, i.e. the relative abundance of rodent, bird and reptile hosts. Indeed the different *B. burgdorferi* s.l. genospecies are associated with different reservoir hosts. For example, *B. afzelii* and *B. bavariensis* are rodent specialists, *B. garinii* and *B. valaisiana* are bird specialists, and *B. burgdorferi* s.s. is a generalist that can infect birds and rodents (reviewed in [21]). Alternatively, strong bottlenecks within sites could lead to the local dominance of specific genospecies that may vary over time (e.g. [43, 44]).

We found no evidence that *B. burgdorferi* s.l. prevalence is higher at the range margins of *I. ricinus* (i.e. at higher elevations), indicating that *B. burgdorferi* s.l.-mediated modifications of *I. ricinus* physiology and behaviour observed in the laboratory ([26, 27], and references therein) plays a minor role in the ongoing colonisation process of marginal habitats in the wild. Rather, *I. ricinus* nymphs at the range margin had substantially lower *B. burgdorferi* s.l. prevalence. This finding is in line with what was found in the Italian Alps, where higher *Borrelia* prevalence was observed at locations < 1400 m.a.s.l compared to > 1400 m.a.s.l (note that ticks were found up to > 1800 m.a.s.l at these study sites) [41]. Our analytical approach allows us to describe changes in *Borrelia* prevalence with elevation in more detail and highlights that the patterns we observe are more consistent with a sudden decrease above a certain elevation, with reduced *Borrelia* prevalence specifically at the range margin, rather than a gradual decline with increasing elevation across the entire tick range. Indeed, analyses within the core range of ticks revealed no association between *Borrelia* prevalence and elevation.

The observed patterns may be due to different non-exclusive processes. The colder microclimate at high elevations may decrease the survival of *B. burgdorferi* s.l. in *I. ricinus* and slow down its multiplication and transmission. Effects of meteorological temperature on pathogen development (i.e. the development from the ingested infectious stage to the stage in the salivary glands) and transmission efficiency have been observed in malaria-mosquito [45, 46] and trypanosome-tsetse fly systems (references in [47]). Although protozoan development is not directly comparable to bacterial multiplication, many bacteria similarly utilize the environmental temperature as a signal to determine their location and to regulate expression of a large set of proteins necessary for survival, multiplication, migration and transmission [48, 49]. For example, the expressions of Outer Surface Protein A and C of *B. burgdorferi* s.l., which are involved in its survival in the tick midgut and its dissemination into the tick salivary glands or into its vertebrate host, appear to be mediated by differences in ambient temperature [50]. To our knowledge, no study evaluated the effect of meteorological temperature on the expression of vector-specific or host-specific proteins, or the expression of virulence genes in *B. burgdorferi* s.l.. Although temperature and other abiotic factors are often involved to explain the lower pathogen prevalence at higher elevations [51], the lack of a gradual decline in *Borrelia* prevalence with increasing elevation within the core range of ticks makes it unlikely that such factors are the sole explanation for the low *Borrelia* prevalence at range margins.

Alternatively, the pattern may also be affected by other processes such as changes in host community, host species abundance, habitat quality or population structure of hosts/vectors in marginal habitats. Indeed, Patot et al. (2010) found no effect of temperature on filamentous virus transmission efficiency, but observed a clear relationship between virus prevalence and the density of its host, a parasitoid wasp. Similarly, *B. burgdorferi* s.l. transmission may be lower in *I. ricinus* populations at the range margin that are more fragmented [52], less dense [17] or/and less genetically diverse [53] than populations at the core range. Similar processes may act on the reservoir host populations of *B. burgdorferi* s.l. (e.g. rodents and birds). However detailed knowledge about the composition of the host community, host abundance, *I. ricinus* infestation of these host populations as well as *B. burgdorferi* s.l. prevalence would be necessary to link host abundance and *B. burgdorferi* s.l. prevalence in questing *I. ricinus*.

Finally, because edge populations are usually founded by only few individuals, stochastic processes may lead to the loss of pathogens in ticks at the range margins. Thus, the combination of stochastic events and host and vector population structure that hinder *B. burgdorferi* s.l. transmission may lead to local extinctions of *B. burgdorferi* s.l. at the range edges (e.g. [18]). It is often assume that vertebrate hosts dispersing ticks on wide ranges are mainly birds and wild cervids (e.g. red deers and chamois). One may argue that because the latter are incompetent hosts for *Borrelia* spp., it may dilute infection prevalence in ticks at the range margins. The lower *Borrelia* infection prevalence at the range margins supports this hypothesis. However a sudden drop in *Borrelia* prevalence above a certain elevation would also be consistent with a higher mortality of ticks driven by abiotic factors. Moreover the dominance of *B. afzelii* in this study, and in the Italian Alps [41], suggests that small rodents may play a particularly important role in the maintenance of *Borrelia* infection along elevational clines in the Alps. Though, further investigations are needed to evaluate these hypotheses in detail.

Our study is correlational and has limitations. First, fewer ticks were collected at the range margin, which will decrease the precision of the prevalence estimate at these sites. Second, a time-based approach (which is less reliable than an area-based approach) was used to estimate questing tick abundance because of the rugged mountain terrain of the study area. Third, vegetation structure varies substantially across the elevation range and could affect tick detection despite a consistent sampling effort. However, the decrease of *I. ricinus* abundance above 1150 m.a.s.l. was observed in different life-stages (Lemoine pers. obs.), which have different questing behaviours and biological needs. Therefore, we are confident that the decrease of *I. ricinus* abundance above 1150 m.a.s.l. is not a methodological artefact but describes an ecological pattern. Fourth, our approach does not allow to disentangle between range-expanding ticks bringing the pathogen with them (i.e., dispersal to a novel habitat) and the enzootic cycle being less likely to become established (i.e., establishment in a novel habitat, for example infecting less dense host populations). Fifth, whereas numerous studies in Europe and North America have documented a range expansion of ticks to higher elevations and latitudes due to climate change (reviewed in [19]), we do not have the long-term monitoring data to directly demonstrate this range expansion at our study sites. Finally, the presence of questing *I. ricinus* ticks in the vegetation does not prove that *I. ricinus* populations are established locally, or will survive through the winter. High elevation habitats might thus represent a ‘ragged edge’ rather than an expansion front. Ultimately, an experimental approach, in which the *B. burgdorferi* s.l. infection status of *I. ricinus* is manipulated and the colonisation of marginal habitats is monitored would be required to test conclusively whether *B. burgdorferi* s.l. facilitates tick range expansion to marginal habitats in the wild. But as stated previously, this would be an enormous practical challenge.

## Conclusions

Overall, we found no evidence that *B. burgdorferi* s.l.-induced changes in *I. ricinus* behaviour or physiology that facilitate *I. ricinus* range expansion to higher elevations in the Swiss Alps. Rather, questing *I. ricinus* in marginal habitats are less likely to carry *B. burgdorferi* s.l.. These findings show that when bitten by a tick, the risk of human *B. burgdorferi* s.l. infection is lower, rather than higher, in regions where *I. ricinus* is newly emerging. Low *B. burgdorferi* s.l. prevalence at *I. ricinus* range margins may enhance population growth and competitive ability of hosts and vectors. Less infected, hosts may, for example, invest differently in immunity and reproduction than hosts in core populations [15, 54], which can affect host-parasite interactions when the parasite finally invades host populations at the range margins [55]. A better understanding of eco-evolutionary processes between pathogens, vectors and hosts at range margins, and their effect on pathogen life-history and virulence evolution, will therefore be a fruitful next step (e.g. [55]), and will contribute to a better prediction of zoonotic disease risks in regions where vectors and pathogens are newly emerging due to climate change.

## Methods

### Study area

We sampled questing *I. ricinus* at fifteen sites across an elevational range from 528 to 1774 m.a.s.l. in the Swiss Alps (Kanton Graubünden, Fig. 1, Table 1). Each site was visited three times, once in June, July and August 2014 to obtain a measure of questing tick abundance per site. During each dragging event, four people dragged a white blanket (1 m × 1 m each) slowly over the ground vegetation during 15 min each. During these 15 min, each collector regularly checked for ticks on their blanket and transferred them to Eppendorf tubes containing 95% ethanol. The count of each collector was pooled to provide an estimate of questing tick abundance per dragging event per hour (Table 2). Although an area-based estimation is often more accurate than a time-based approach, it was not possible to apply such a method in our study because of the rugged mountain terrain of the Swiss Alps. Tick life stage and species were verified with a dissection microscope in the laboratory on the basis of morphologic features following [56].

*Borrelia burgdorferi* s.l. infection in questing *Ixodes ricinus* nymphs.

We randomly selected 34–35 questing *I. ricinus* nymphs per site for *B. burgdorferi* s.l. detection. When fewer than 35 nymphs were collected at a site (Table 1), additional questing nymphs were collected for *Borrelia* detection one day after estimating questing tick abundance. All collected nymphs were analysed when fewer than 35 nymphs were available (Table 1). DNA was extracted using the ‘HotShot’ method [57, 58] with slight modifications. Each nymph was incubated with 120 μL of alkaline lysis reagent (25 mM NaOH, 0.2 mM EDTA) and a metal bead (2 mm in diameter) at 95 °C for 15 min. After homogenisation with a Retsch TissueLyser (Haan, Germany) for 2 min, the samples were incubated at 95 °C for 15 min. After cooling, 60 μL 40 mM Tris–HCl was added to each tube.

To assess the *B. burgdorferi* s.l. infection status of *I. ricinus* nymphs, we used a combination of two complimentary approaches. First, we specifically targeted *B. afzelii,* the most common *Borrelia* genospecies at the study sites [54]*,* using a highly sensitive quantitative real-time PCR (qPCR) assay using the *B. afzelii*-specific primers Fla5F 5′-CACCAGCATCACTTTCAGGA-3′ and Fla6R 5′-CTCCCTCACCAGCAAAAAGA-3′ [57] on a StepOnePlus Real-Time PCR machine (Applied Biosystems). Each reaction contained 10 μL of SYBR Select Master Mix (Applied Biosystems), 0.8 μL of each primer (10 μM), 4.4 μL of water and 4 μL of template DNA in a final volume of 20 μL. Two series of four standards, two negative and two positive tick samples, as well as two no-template controls were included in each run. The PCR amplification protocol consisted of an initial denaturation step at 50 °C and 95 °C for 2 min each, followed by 42 cycles of 95 °C for 15 s, 59 °C for 30 s, and 72 °C for 30 s. The length of the amplicon was 129 bp and the melting temperature was between 77.3 °C and 77.9 °C. A 100% repeatability was obtained based on 40 samples (10 positives and 30 negatives) when amplification occurred before 34 cycles. Out of 85 *B. afzelii-*positive samples, only two samples amplified between 34 and 37 cycles. These samples were repeated and considered to be *B. afzelii*-infected only if they were found to be positive twice.

In a second step, all samples that were found to be *B. afzelii*-negative using the qPCR approach described above were screened for the presence of other *B. burgdorferi* s.l*.* genospecies using 16S-LD primers designed to target all *Borrelia* genospecies associated with Lyme borreliosis [59]. Amplifications were performed in a total volume of 10 μL containing 0.2 μL JumpStart Taq DNA Polymerase (Sigma-Aldrich), 0.5 μL of each primer (300 nM; LD-F: 5′-ATGCACACTTGGTGTTAACTA-3′ and LD-R: 5′-GACTTATCACCGGCAGTCTTA-3′) and 2 μL of DNA template. The PCR amplification protocol consisted of an initial denaturation step at 94 °C for 1 min, followed by 42 cycles of denaturation at 94 °C for 30 s, annealing at 50 °C for 30 s, and extension at 72 °C for 90 s, with a final elongation step at 72 °C for 10 min. PCR products were visualized under UV light on 1% agarose gels that were stained with SYBR^®^ Safe DNA gel stain (Thermo Scientific). The length of the amplicon was 351 bp. We used DNA from six reference genospecies B31 and Pbre (*B. burgdorferi* s.s), Phei (*B. garinii*), Pko and PVPM (*B. afzelii*) and Pbi (*B. bavariensis*) to verify that they are detected with the 16S approach. All references were successfully amplified.

Each run included *B. afzelii*-positive samples identified with the qPCR (see above) as controls. All of these samples were found to be *B. burgdorferi* s.l.-positive using the 16S method. The repeatability based on 39 samples (12 negative and 27 positive samples) was 85%. Out of the six samples that were not repeatable with the 16S method, three were previously quantified with the qPCR method and were found to have very low *B. afzelii* DNA concentrations (i.e. more than 30 cycles on the qPCR) suggesting that the sensitivity of the 16S approach decreases at low infection intensities. During the development of the methods, some fragments were sequenced and all were found to be *B. burgdorferi* s.l. suggesting that both methods were specific to the Lyme borreliosis causing genospecies.

### Statistical analyses

Questing *I. ricinus* abundance (Table 2) was analysed using a generalized mixed effect model with the negative binomial distribution (with the parameterization σ = μ(1 + μ/k)) and site ID was included as a random effect. Preliminary analyses showed that the negative binomial distribution (with the parameterization σ = μ(1 + μ/k)) described the abundance of nymphs (or nymphs and adults combined) best among the following distributions: Poisson, negative binomial (with the parameterization σ = μ(1 + μ/k) or σ = φμ) with or without zero-inflation (results not shown). We first tested for an association between tick abundance across life stages (nymphs and adults) and elevation or season. To this end, we included elevation and elevation^2^ in interaction with tick stage, or in interaction with sampling session as fixed effects (i.e. as explanatory variables to quantify an average effect, *β*) in the mixed model. Finally, we defined the range margin based on questing tick abundance (see Results) and included range type (range core (C) vs range margin (M)) of each site as a fixed effect.

*Borrelia* infection (i.e. *B. burgdorferi* s.l. or *B. afzelii* infection) in *I. ricinus* nymphs (Table 2) was analysed using a generalized mixed effect model with a binomial error structure. The number of infected and non-infected nymphs per dragging event was used as the dependant variable and site ID was included as a random effect. In a first step, we tested for an association between *B. burgdorferi* s.l. or *B. afzelii* prevalence and elevation by including elevation and elevation^2^ as fixed effects (i.e. as explanatory variables to quantify an average effect, *β*) in the mixed model. In a second step, we tested if *B. burgdorferi* s.l. or *B. afzelii* prevalence was higher at the range margin of *I. ricinus* (i.e. at high elevations). To this end, we compared the *B. burgdorferi* s.l. or *B. afzelii* prevalence in *I. ricinus* at the range margin (sites > 1400 m.a.s.l.) to *B. burgdorferi* s.l. or *B. afzelii* prevalence in the core range of *I. ricinus* (sites < 1150 m.a.s.l.) by including range type (range core (C) vs range margin (M)) of each site as a fixed effect.

Finally, we focused on the patterns within the core range of *I. ricinus* by investigating the association between *B. burgdorferi* s.l. or *B. afzelii* prevalence and elevation, and the relationship between *B. burgdorferi* s.l. or *B. afzelii* prevalence and questing *I. ricinus* abundance within the core range of *I. ricinus*. The effect of questing *I. ricinus* abundance was investigated by including questing *I. ricinus* abundance and questing *I. ricinus* abundance^2^ as fixed effects. Site ID was again included as a random effect.

To ensure independence between main and quadratic terms, all continuous variables were standardized (by subtracting the population mean from each sample and dividing it by the standard deviation). The significance of explanatory variables was assessed using likelihood ratio tests. The significance of the linear terms was assessed after removing the non-significant quadratic terms. Statistical analyses were performed using the package glmmADMB [60, 61] in R 3.2.3 [62].

## Data Availability

The dataset supporting the conclusions of this article is included within the article and its additional files.

## Abbreviations

m.a.s.l.: Metres above mean sea level
*B.*: *Borrelia*
*I.*: *Ixodes*
s.l.: Sensu lato
s.s.: Sensu stricto
spp.: Species
DNA: Deoxyribonucleic acid
EDTA: Ethylenediaminetetraacetic acid
PCR: Polymerase chain reaction
UV: Ultraviolet
ID: Identifier
C: Range core
M: Range margin
O: Outside of the range
UNT: Untevaz
MAL: Malans
ROD: Rodels
SAG: Sagogn
PAS: Passug
TRI: Trimmis
BON: Bonaduz
CAS: Castiel
SEE: Seewis
FLI: Flims
TOM: Tomils
RUS: Ruschein
PRA: Praden
FEL: Feldis
VIL: Vilan

## References

[1] Price PW (1980). Evolutionary biology of parasites.

[2] Poulin R (1994). The evolution of parasite manipulation of host behaviour: a theoretical analysis. Parasitology.

[3] Thomas F, Schmidt-Rhaesa A, Martin G, Manu C, Durand P, Renaud F (2002). Do hairworms (Nematomorpha) manipulate the water seeking behaviour of their terrestrial hosts?. J Evol Biol.

[4] Dobson AP (1988). The population biology of parasite-induced changes in host behavior. Q Rev Biol.

[5] Hurd H (2003). Manipulation of medically important insect vectors by their parasites. Annu Rev Entomol.

[6] Lefevre T, Thomas F (2008). Behind the scene, something else is pulling the strings: emphasizing parasitic manipulation in vector-borne diseases. Infect, Genet Evol.

[7] Cezilly F, Favrat A, Perrot-Minnot MJ (2013). Multidimensionality in parasite-induced phenotypic alterations: ultimate versus proximate aspects. J Exp Biol.

[8] Thomas F, Adamo S, Moore J (2005). Parasitic manipulation: where are we and where should we go?. Behav Processes.

[9] Britton JR, Andreou D (2016). Parasitism as a driver of trophic niche specialisation. Trends Parasitol.

[10] Dunn AM, Torchin ME, Hatcher MJ, Kotanen PM, Blumenthal DM, Byers JE (2012). Indirect effects of parasites in invasions. Funct Ecol.

[11] Wood CL, Johnson PTJ (2015). A world without parasites: exploring the hidden ecology of infection. Front Ecol Environ.

[12] Hatcher MJ, Dick JTA, Dunn AM (2014). Parasites that change predator or prey behaviour can have keystone effects on community composition. Biol Lett.

[13] Heger T, Jeschke JM (2014). The enemy release hypothesis as a hierarchy of hypotheses. Oikos.

[14] Kolar CS, Lodge DM (2001). Progress in invasion biology: predicting invaders. Trends Ecol Evol.

[15] White TA, Perkins SE (2012). The ecoimmunology of invasive species. Funct Ecol.

[16] Briers RA (2003). Range limits and parasite prevalence in a freshwater snail. Proc R Soc Lond Ser B Biol Sci.

[17] Patot S, Martinez J, Allemand R, Gandon S, Varaldi J, Fleury F (2010). Prevalence of a virus inducing behavioural manipulation near species range border. Mol Ecol.

[18] Phillips BL, Kelehear C, Pizzatto L, Brown GP, Barton D, Shine R (2010). Parasites and pathogens lag behind their host during periods of host range advance. Ecology.

[19] Medlock JM, Hansford KM, Bormane A, Derdakova M, Estrada-Pena A, George JC, et al. Driving forces for changes in geographical distribution of *Ixodes ricinus* ticks in Europe. Parasites Vectors. 2013;6. 10.1186/1756-3305-6-1 PMC3549795 23281838

[20] Ostfeld RS, Brunner JL (2015). Climate change and *Ixodes* tick-borne diseases of humans. Philos Trans R Soc Lond Ser B Biol Sci.

[21] Margos G, Vollmer SA, Ogden NH, Fish D (2011). Population genetics, taxonomy, phylogeny and evolution of *Borrelia burgdorferi *sensu lato. Infect, Genet Evol.

[22] Rauter C, Hartung T (2005). Prevalence of *Borrelia burgdorferi *sensu lato genospecies in *Ixodes ricinus* ticks in Europe: a meta analysis. Appl Environ Microbiol.

[23] Hellgren O, Andersson M, Raberg L (2011). The genetic structure of *Borrelia afzelii* varies with geographic but not ecological sampling scale. J Evol Biol.

[24] Comstedt P, Bergström S, Olsen B, Garpmo U, Marjavaara L, Mejlon H (2006). Migratory passerine birds as reservoirs of Lyme borreliosis in Europe. Emerg Infect Dis.

[25] McCoy KD, Léger E, Dietrich M (2013). Host specialization in ticks and transmission of tick-borne diseases: a review. Front Cell Infect Microbiol.

[26] Herrmann C, Gern L (2015). Search for blood or water is influenced by *Borrelia burgdorferi* in *Ixodes ricinus*. Parasit Vectors.

[27] Benelli G (2020). Pathogens manipulating tick behavior-through a glass, darkly. Pathogens.

[28] Gassner F, Hartemink N. Tick–Borrelia interactions: burden or benefit? Ecology of parasite-vector interactions: Springer; 2013. p. 141-54.

[29] Herrmann C, Gern L (2013). Survival of *Ixodes ricinus* (Acari: Ixodidae) nymphs under cold conditions is negatively influenced by frequent temperature variations. Ticks Tick Borne Dis.

[30] Madhav NK, Brownstein JS, Tsao JI, Fish D (2004). A dispersal model for the range expansion of blacklegged tick (Acari: Ixodidae). J Med Entomol.

[31] Aeschlimann A, Chamot E, Gigon F, Jeanneret J-P, Kesseler D, Walther C (1987). *B. burgdorferi* in Switzerland. Zentralblatt für Bakteriologie, Mikrobiologie und Hygiene.

[32] Jouda F, Perret JL, Gern L (2004). *Ixodes ricinus* density, and distribution and prevalence of *Borrelia burgdorferi* sensu lato infection along an altitudinal gradient. J Med Entomol.

[33] Moran Cadenas F, Rais O, Jouda F, Douet V, Humair PF, Moret J (2007). Phenology of *Ixodes ricinus* and infection with *Borrelia burgdorferi* sensu lato along a north- and south-facing altitudinal gradient on Chaumont Mountain, Switzerland. J Med Entomol.

[34] Gern L, Moran Cadenas F, Burri C (2008). Influence of some climatic factors on *Ixodes ricinus* ticks studied along altitudinal gradients in two geographic regions in Switzerland. Int J Med Microbiol.

[35] James MC, Bowman AS, Forbes KJ, Lewis F, McLeod JE, Gilbert L (2013). Environmental determinants of *Ixodes ricinus* ticks and the incidence of *Borrelia burgdorferi* sensu lato, the agent of Lyme borreliosis, in Scotland. Parasitology.

[36] Stünzner D, Hubálek Z, Halouzka J, Wendelin I, Sixl W, Marth E (2006). Prevalence of *Borrelia burgdorferi* sensu lato in the tick *Ixodes ricinus* in the Styrian mountains of Austria. Wien Klin Wochenschr.

[37] Ragagli C, Mannelli A, Ambrogi C, Bisanzio D, Ceballos L, Grego E (2016). Presence of host-seeking *Ixodes ricinus* and their infection with *Borrelia burgdorferi* sensu lato in the Northern Apennines. Italy Exp Appl Acarol.

[38] Danielová V, Daniel M, Schwarzová L, Materna J, Rudenko N, Golovchenko M (2010). Integration of a tick-borne encephalitis virus and *Borrelia burgdorferi* sensu lato into mountain ecosystems, following a shift in the altitudinal limit of distribution of their vector, *Ixodes ricinus* (Krkonoše mountains, Czech Republic). Vector-Borne Zoonotic Dis.

[39] Daniel M, Materna J, Hönig V, Metelka L, Danielová V, Harcarik J (2009). Vertical distribution of the tick *Ixodes ricinus* and tick-borne pathogens in the northern Moravian mountains correlated with climate warming (Jeseníky Mts., Czech Republic). Central Eur J Public Health..

[40] Danielová V, Rudenko N, Daniel M, Holubová J, Materna J, Golovchenko M (2006). Extension of *Ixodes ricinus* ticks and agents of tick-borne diseases to mountain areas in the Czech Republic. Int J Med Microbiol.

[41] Garcia-Vozmediano A, Krawczyk AI, Sprong H, Rossi L, Ramassa E, Tomassone L (2020). Ticks climb the mountains: *Ixodid* tick infestation and infection by tick-borne pathogens in the Western Alps. Ticks Tick-borne Dis.

[42] Herrmann C, Gern L, Voordouw MJ (2013). Species co-occurrence patterns among Lyme Borreliosis pathogens in the tick vector *Ixodes ricinus*. Appl Environ Microbiol.

[43] Bruyndonckx N, Henry I, Christe P, Kerth G (2009). Spatio-temporal population genetic structure of the parasitic mite *Spinturnix bechsteini* is shaped by its own demography and the social system of its bat host. Mol Ecol.

[44] Criscione CD, Blouin MS (2006). Minimal selfing, few clones, and no among-host genetic structure in a hermaphroditic parasite with asexual larval propagation. Evolution.

[45] Eling W, Hooghof J, van de Vegte-Bolmer M, Sauerwein R, Van Gemert G, editors. Tropical temperatures can inhibit development of the human malaria parasite *Plasmodium falciparum* in the mosquito. Proceedings of the Section Experimental and Applied Entomology of the Netherlands Entomological Society (N. E. V.) Amsterdam; 2001.

[46] Noden B, Kent M, Beier JC (1995). The impact of variations in temperature on early *Plasmodium falciparum* development in *Anopheles stephensi*. Parasitology.

[47] Moore S, Shrestha S, Tomlinson KW, Vuong H (2012). Predicting the effect of climate change on African trypanosomiasis: integrating epidemiology with parasite and vector biology. J R Soc Interface.

[48] Miller JF, Mekalanos JJ, Falkow S (1989). Coordinate regulation and sensory transduction in the control of bacterial virulence. Science.

[49] Steinmann R, Dersch P (2013). Thermosensing to adjust bacterial virulence in a fluctuating environment. Future Microbiol.

[50] Schwan TG, Piesman J (2002). Vector interactions and molecular adaptations of Lyme disease and relapsing fever spirochetes associated with transmission by ticks. Emerg Infect Dis.

[51] Lafferty KD (2009). The ecology of climate change and infectious diseases. Ecology.

[52] Hanski I (1999). Metapopulation ecology.

[53] Sexton JP, McIntyre PJ, Angert AL, Rice KJ. Evolution and ecology of species range limits. 2009.

[54] Cornetti L, Hilfiker D, Lemoine M, Tschirren B (2018). Small-scale spatial variation in infection risk shapes the evolution of a *Borrelia* resistance gene in wild rodents. Mol Ecol.

[55] Kelehear C, Brown GP, Shine R (2012). Rapid evolution of parasite life history traits on an expanding range-edge. Ecol Lett.

[56] Hillyard PD. Ticks of north-west Europe: Field Studies Council; 1996.

[57] Råberg L (2012). Infection intensity and infectivity of the tick-borne pathogen *Borrelia afzelii*. J Evol Biol.

[58] Truett GE, Heeger P, Mynatt RL, Truett AA, Walker JA, Warman ML (2000). Preparation of PCR-quality mouse genomic DNA with hot sodium hydroxide and tris (HotSHOT). Biotechniques.

[59] Marconi RT, Garon CF (1992). Development of polymerase chain reaction primer sets for diagnosis of Lyme disease and for species-specific identification of Lyme disease isolates by 16S rRNA signature nucleotide analysis. J Clin Microbiol.

[60] Fournier DA, Skaug HJ, Ancheta J, Ianelli J, Magnusson A, Maunder M (2012). AD Model Builder: using automatic differentiation for statistical inference of highly parameterized complex nonlinear models. Optim Methods Softw..

[61] Skaug HJ, Fournier DA, Bolker B, Magnusson A, Nielsen A. Generalized linear mixed models using AD model builder. R package version 0.8.0. 2014.

[62] R Core Team. R: A language and environment for statistical computing. R Foundation for Statistical Computing. Vienna, Austria: R Foundation for Statistical Computing; 2013.

